# Phrenic Artery Hemorrhage after Percutaneous Portal Vein Stenting to Treat Cavernous Transformation Following Living Donor Liver Transplantation: A Case Report

**DOI:** 10.1155/2011/481237

**Published:** 2011-06-27

**Authors:** Qiang Huang, Ningning Lu, Renyou Zhai

**Affiliations:** Beijing Chao-Yang Hospital, Capital Medical University, Beijing 100020, China

## Abstract

Cavernous transformation is a condition which has an acutely developing harmful effect over intestinal circulation compromising the patients' life before development of portal hypertension and its results. We present the case of phrenic artery hemorrhage after successful percutaneous portal vein stenting to treat cavernous transformation following LDLT. The patient survived the hazard complication with prompt surgery. Three factors may be related to the rare complication in the case were analyzed, including affluent new vessels around the diaphragm related to LDLT procedure, high puncture site allowing the diaphragm been injured, and anticoagulation given before the puncture and soon after the procedure. Cautions should be taken for the interventional procedures in this extreme condition. Cavernous transformation is a condition which has an acutely developing harmful effect over intestinal circulation compromising the patients' life before development of portal hypertension and its results (see the work of Harmanci and Bayraktar (2007)). It is even worse when this happens in a patient after LDLT (living donor liver transplantation). Herein we have presented a case of phrenic artery hemorrhage after successful percutaneous portal vein stenting to treat cavernous transformation following LDLT. The patient survived the hazard complication with prompt surgery.

## 1. Case Report

**Figure 1:**
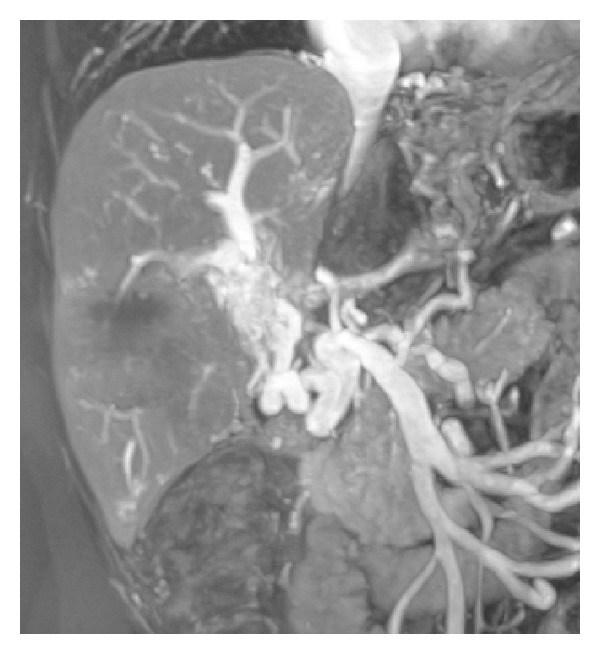
MR angiography showed PVAS with main portal vein occluded completely.

A 24-year-old female received LDLT for liver failure caused by hepatitis-B-related cirrhosis in March 2007. Four months after LDLT, PVAS (portal vein anastomotic stenosis) was detected during routine color Doppler ultrasonography screening. An MRI (magnetic resonance imaging) was performed, and the diagnosis of PVAS was confirmed, thereafter she underwent portal vein balloon dilation at local hospital. But PVAS was confirmed again seven months later. No treatment was attempted because the blood flow still existed in the severe stenotic portal vein and no obvious clinical symptom developed. Twenty days ago, the patient suffered an episode of esophageal and gastric varices bleeding which was controlled with conservative therapy. Another MR angiography showed PVAS with main portal vein occluded completely (Figure 1). She was referred to our section for a portal vein angioplasty or stenting.

## 2. Interventional Procedure

**Figure 2:**
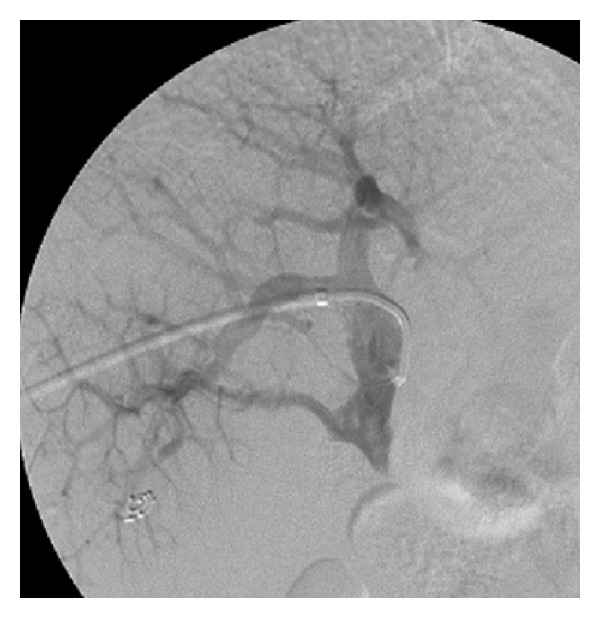
Initial portography revealed completely occluded main portal vein with PVAS and esophageal and gastric varices.

**Figure 3:**
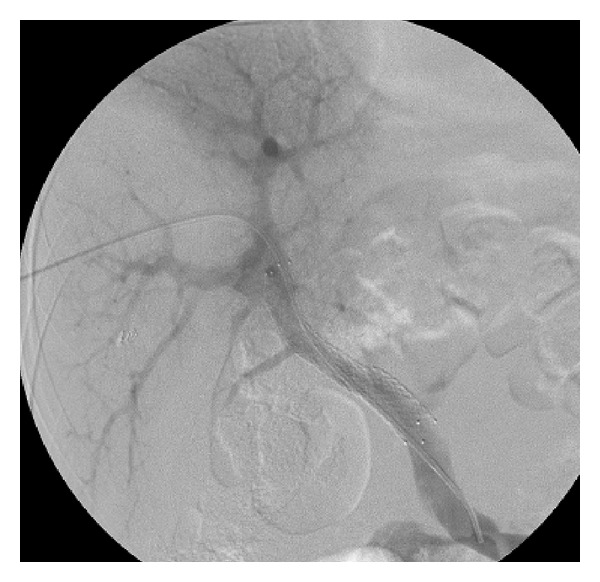
Poststenting portography showed portal venous patency and inflow had recovered completely with the gastroesophageal varices almost invisible.

Informed consent was obtained before the interventional procedure. The patient underwent the procedure under local anesthesia. Anticoagulation was given with 6250 u heparin as a single bolus. A percutaneous transhepatic puncture of the intrahepatic portal vein was performed using a 21-gauge Chiba needle via a right-sided intercostal approach under fluoroscopic guidance. The intrahepatic portal vein was successfully displayed with the first puncture. Portography was obtained with standard catheter and guidewire exchange technique. Initial portography revealed completely occluded main portal vein with PVAS and esophageal and gastric varices (Figure 2). A 0.035-inch guidewire and a Kumpe catheter were then used to traverse the occluded portal vein successfully. The remaining stenotic segment was dilated with a balloon (8 mm in diameter and 4 cm in length). A self-expandable metallic stent (10 mm in diameter and 6 cm in length) was deployed to cover the stenosis. Poststenting portography (Figure 3) was obtained repeatedly until a satisfactory result, which showed that portal venous patency and inflow had recovered completely with the gastroesophageal varices almost invisible, had been achieved. Then the catheter was removed, and the puncture tract was embolized with compressed Gelfoambars. Dalteparin sodium of 5000 u was administrated once with subcutaneous injection after stent implantation. 

 The patient experienced an uneventful postprocedure outcome. She developed anxiety and hypotension soon after being back in the ward. Emergency thorax CT revealed large amount of hematothorax. Thoracotomy was immediately performed, and phrenic artery hemorrhage was confirmed after large amount of pleural effusion and blood clots were aspirated. The blooding point was mended and hemorrhage stopped. The patient recovered after spending 4 days in the intensive care unit. A color Doppler ultrasonography showed patent portal vein. The patient was discharged 7 days after surgery and remained well during the follow-up thereafter.

## 3. Discussion

 Cavernous transformation is a condition that has acutely developing harmful effects over intestinal circulation compromising the patients' life before development of portal hypertension and its results [1]. There is no report on percutaneous portal vein stenting to treat cavernous transformation following LDLT to our knowledge. Here we present a successful portal vein stenting for cavernous transformation following LDLT while a rare complication of hemorrhage was encountered. 

 The majority of patients who are found to have portal vein stenosis or cavernous transformation are asymptomatic and detected on routine screening ultrasound [2], just as this case. Percutaneous treatment of portal vein complications after LDLT is a routine and mature technique in the literatures [3, 4]. No particular severe complication related to the interventional procedure was reported in the literature. 

 Three factors may be related to the rare complication in the case according to our own analysis, the first of which is affluent new vessels around the diaphragm seen during the thoracotomy. This new vessels might come from inflammatory reaction after liver transplantation. The second factor is that the puncture site may be too high to allow the Chiba needle to hurt the diaphragm. Although the intrahepatic portal vein was successfully displayed with the first puncture, this injury is consistent with the ruptured phrenic artery revealed during the surgery. The third factor may be anticoagulation given before the puncture and soon after the procedure. There is variability with the use of anticoagulation before and after balloon while most literature just did not mention this in detail [2, 3]. No definite conclusion can be drawn now. The anticoagulation may be given too early or with a too high dose to allow the hemorrhage to happen or become worse in this case. 

 In conclusion, percutaneous portal vein stenting may play an important role in treating cavernous transformation following LDLT, but great caution must be taken for the interventional procedures in this extreme condition.
